# The Clinical Significance of Cerebrospinal Fluid Reticulon 4 (RTN4) Levels in the Differential Diagnosis of Neurodegenerative Diseases

**DOI:** 10.3390/jcm10225281

**Published:** 2021-11-13

**Authors:** Agnieszka Kulczyńska-Przybik, Maciej Dulewicz, Agnieszka Słowik, Renata Borawska, Alina Kułakowska, Jan Kochanowicz, Barbara Mroczko

**Affiliations:** 1 Department of Neurodegeneration Diagnostics, Medical University of Bialystok, 15-269 Bialystok, Poland; maciej.dulewicz@umb.edu.pl (M.D.); renata.borawska@umb.edu.pl (R.B.); mroczko@umb.edu.pl (B.M.); 2 Department of Neurology, Jagiellonian University, 30-688 Kraków, Poland; slowik@neuro.cm-uj.krakow.pl; 3 Department of Neurology, Medical University of Bialystok, 15-269 Bialystok, Poland; alina.kulakowska@umb.edu.pl (A.K.); jan.kochanowicz@umb.edu.pl (J.K.); 4 Department of Biochemical Diagnostics, Medical University of Bialystok, 15-269 Bialystok, Poland

**Keywords:** RTN-4A, reticulons, biomarkers, Alzheimer’s disease, Parkinson’s disease, multiple sclerosis, neurodegenerative diseases

## Abstract

Neurodegenerative diseases (NDs) belong to the top global causes of mortality. Diagnostic approaches to improve early diagnosis and differentiation of these diseases are constantly being sought. Therefore, we aimed to assess the cerebrospinal fluid (CSF) concentrations of Reticulon 4 (RTN4) in patients with neurodegenerative diseases and evaluate the potential clinical usefulness of this protein. RTNs are transmembrane proteins mediating neuroanatomical plasticity and functional recovery after central nervous system injury or diseases. According to our best knowledge, this is the first investigation providing the data concerning the dynamic of CSF RTN4 protein levels in patients with different NDs. Methods: Overall, 77 newly diagnosed patients with neurodegenerative diseases, including Alzheimer’s disease (AD), Parkinson’s disease (PD), and multiple sclerosis (MS), as well as 21 controls, were enrolled in the study. The CSF concentrations of tested proteins were assessed using immunological assays. Results: We revealed significantly higher CSF RTN4A levels in patients with AD, PD, and MS in comparison to the controls. Moreover, the comparative analysis of RTN4 concentration between different neurodegenerative diseases revealed the highest concentration of RTN4A in AD patients and a statistically significant difference between AD vs. PD, and AD vs. MS groups. The increased CSF level of the protein correlated with Tau, and pTau181 proteins in AD as well as in PD patients. Conclusions: Our study presents a previously not identified clinical utility of RTN4 in the differential diagnosis of neurodegenerative diseases.

## 1. Introduction

Neurodegenerative diseases (NDs) are the most common cause of cognitive impairment and a growing cause of morbidity and mortality worldwide, particularly in older adults. NDs are still incurable, difficult to detect them at earlier stages due to the long asymptomatic incubation period, and hard to differentiate at later stages due to the similarities of different NDs or their atypical forms [1,2]. Therefore, it is important to discriminate and diagnose these disorders accurately [3]. Moreover, available diagnostic tests are still insufficient [4]. Therefore, biomarkers that might improve the diagnosis of these diseases are urgently sought. At present, there are no universally accepted biomarkers with confirmed reliability as a measure of disease burden in NDs, except CSF biomarkers for Alzheimer’s disease, which is a significant problem in clinical practice [3]. So far, many investigations of potential biomarkers have been carried out in CSF of patients with ND. For example, cerebrospinal fluid (CSF) biomarkers associated with cognitive performance which may reflect neurodegeneration (T-tau, NFL, NSE, VILP-1), APP metabolism (Aβ42, Aβ40, Aβ38, sAPPα, and sAPPβ), tangle pathology (P-tau), glial activation (YKL-40, MCP-1, and GFAP), and synaptic dysfunction (neurogranin, SNAP-25, GAP43) were identified in AD [5,6]. Moreover, potential CSF biomarkers related to the pathomechanism of MS include markers of demyelination (MBP—*myelin basic protein*, MBP-LM, anti-MBP, anty-MOG, anti-PLP), glial activation (S100b, GFAP), axonal damage (Tau protein, NF, NSE—*Neuron-Specific Enolase*, NAA—*N-acetylaspartate*, 14-3-3), and inflammation (CXCL13, IFN-λ, TNF-α, TNF-β, neopterin, soluble CD14, IL-2, IL-2R, IL-6, IL-10) [7,8]. The CSF biomarkers seem to be one of the primary laboratory tools and should be part of these disorders’ routine diagnostics [9].

**Figure 1:**
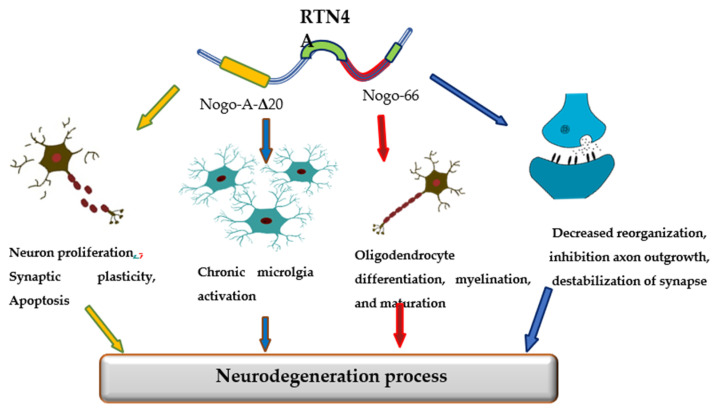
The role of Reticulon 4 (RTN4) in neurodegenerative diseases pathology.

The promising candidate biomarker for neurodegeneration seems to be Reticulon 4 (RTN-4A), also referred to as Nogo-A protein, a neuron-specific protein released from damaged brain cells into the cerebrospinal fluid (CSF). The RTN-4 is one of the inhibitors associated with CNS myelin and belongs to the RTN family [10]. Among four principal RTN family members (RTN-1, -2, -3, -4), the most significant in CNS appears to be RTN-4. RTN-4 consists of two inhibitory domains, carboxy-terminal domain, which is common to reticulons and N-terminal that is unique to Nogo-A, between the two transmembrane stretches, there is a 66-amino-acid loop (Nogo-66) necessary for inhibition of axon growth [10]. Mounting evidence has suggested that reticulons (RTNs) are pivotal factors regulating some physiological as well as pathological processes in the central nervous system. The primary function of RTN-4 is impairment neurite outgrowth and limitation of axonal growth following injury or disease of CNS. However, reticulons regulate a wide range of CNS cells functions, including the development and maintenance of neurons and oligodendrocytes, axonal growth, myelination, synaptic plasticity, and apoptosis [11,12,13]. Additionally, the RTN4A is also engaged in the development of neuroinflammation. The overexpression of RTN4A leads to upregulation of some pro-inflammatory proteins, including CHOP, IL-6 and TNF-α [14]. Increased expression of Nogo623-640 or Nogo-66 may activate T and B cell response. Strong Th1 response with a release of pro-inflammatory molecules and antibody targeting of other myelin antigens can lead to inflammation and neurodegeneration [15]. Various investigations have demonstrated the involvement of RTNs in the pathogenesis of neurodegenerative disorders, such as Alzheimer’s disease (AD), multiple sclerosis (MS), or Parkinson’s disease (PD) [16] (Figure 1).

The relationship of RTN4 with the development of AD pathology has been extensively investigated. In vitro and in vivo studies revealed that increased expression of RTN-4A may favor β-amyloid production and deposition in senile plaques [17]. He et al. revealed the co-immunoprecipitation of BACE1 and RTN4 and described RTN4 as a binding partner of BACE1 [18]. Other studies have reported that RTN4A may promote amyloid β production in the brain by regulating the beta-site amyloid precursor protein-cleaving enzyme1 (BACE1) function and APP processing [19]. The main part of the protein responsible for boosting of Aβ40 and Aβ42 production is Nogo-66 [20]. Nogo-66 binds to its receptor (NgR1) and regulates the generation of Aβ, activating BACE1 and increasing APP level via GTPases of the Rho family, RhoA kinase (ROCK), LimK, and cofilin [20]. Moreover, the activation of ROCK pathway accelerated Aβ42 production, while PKC activation decreases the Aβ_42_ concentration [19,20].

The role of RTN4A in PD is not yet well understood. Studies Schawkat et al. revealed that about 50% of dopaminergic neurons in the substantia nigra co-express RTN4-A, which might act as a protecting factor in lesioned dopaminergic neurons overexpressing RTN-4A [21]. The neuroprotective role of RTN4A in PD is presumably not related to the Nogo-66 domain and the NgR1 complex. It appears to be a possible theory that, opposite to the RTN4A protein, the NgR1 complex has a negative regulatory role on the survival of dopaminergic neurons [22]. In line with this notion is the findings of researchers, who showed that inhibition of NgR1 or LINGO-1 caused a significant increase in the survival of dopaminergic neurons [23].

Mounting evidence indicates that RTN4A might be a pivotal causative factor in MS. Considering that the main pathological mechanism in MS are inflammation and demyelination and that RTN-4A and its receptor NgR1 may modulate both processes, it seems crucial and justifiable to know better the role of this protein in the disease progression. Available studies have shown that RTN-4A and its receptor NgR1 may contribute to the pathogenesis of MS by inhibiting axonal and myelin regeneration and regulating the activation of M1/M2 macrophage via MAPK, ER stress, or transcriptional regulators Yap. Moreover, RTN4-A enhances the recruitment of macrophages by up-regulation of the expression of chemokines CCL2 and cytoskeleton arrangement [24]. The increased RTN4 levels were found in brain tissues, cerebrospinal fluid, and blood of patients with multiple sclerosis [25,26,27,28]. Moreover, the presence of the protein was detected in the early stages of the disease, in the advanced and late stage. However, there was no correlation with the patient’s EDSS disability scale [25]. The Nogo-A and proteins related to its receptor NgR1 have attracted attention as a marker of disease activity and a promising therapeutic target for multiple sclerosis [29].

Investigations revealed the accumulation of RTNs aggregates in pathological dystrophic neurites around senile plaques, NFT, Lewy bodies (LB) [30], as well as the chronic active demyelinating lesions in the brain patients with NDs [25,26]. However, in the available literature, there are little data concerning changes in the concentration of RTNs in the fluids of patients with neurodegenerative diseases and their potential clinical usefulness. It is suggested that monitoring of the changes of RTNs levels may reflect pathological conditions ongoing in the brain during diseases. Therefore, the purpose of the present investigation was to measure the RTN-4 levels in cerebrospinal fluid patients with different neurodegenerative diseases and controls and verify a potential clinical usefulness of this protein. According to our best knowledge, this is the first investigation evaluating and comparing the concentrations of RTN4 protein in CSF patients with NDs.

## 2. Materials and Methods

### 2.1. Study Population

The Ethics Committee approved the study of Bialystok University (No. R-I-002/103/2019), and all patients signed an informed consent form prior to any procedure. The study population consisted of 98 subjects with neurological disorders (64 women and 34 men, median of age 66 years), who were divided into four subgroups: patients with AD (n = 20), patients with PD (n = 19), patients with MS (n = 38), and non-demented subjects as a control group (n = 21) (Table 1). Study participants were diagnosed and treated at the Department of Neurology of Jagiellonian University in Kraków, Poland, and the Department of Neurology of Medical University in Bialystok, Poland. Patients undergo clinical evaluations, neurological, psychiatric, and neuropsychological examinations, neuroimaging tests (magnetic resonance imaging/computed tomography), as well as routine blood and CSF screening assessments. All patients included in the study were in the process of receiving a diagnosis. Additionally, we excluded subjects with blood-CSF barrier dysfunction (elevated Q _alb_) from the study due tu avoid the possible influence of increased RTN-4 levels in the blood as a potential source of the alterations of their CSF levels.

In this investigation, the diagnosis of AD was based on the recommendations from the National Institute on Aging—Alzheimer’s Association (NIA-AA) working groups [31]. The exclusion criteria in the AD group were as follows: history or presence of vascular diseases (i.e., stroke, aortic aneurysm, intracranial aneurysm, cerebral hemorrhage, or arteriovenous malformation), clinically significant central nervous system trauma, intracranial tumors and other malignant neoplasm, severe infections or systemic autoimmune disorders as well as neurological diseases and relevant cerebral abnormalities as assessed by magnetic resonance imaging (MRI) and/or computed tomography (CT). Moreover, the presence of metabolic/endocrine disorders and decreased concentrations of folic acid or vitamin B12, as well as alcohol and/or substance abuse or dependence (except nicotine use), constituted exclusion criteria. Deformity of the lumbosacral spine was also a contraindication to lumbar puncture. In addition, to obtain the most accurate clinical diagnosis of AD neuroimaging and neuropsychological tests were combined with neurochemical AD biomarkers (Aβ1-42, tau and pTau181 levels and Aβ1-42/Aβ1-40 ratio values). The concentrations of AD biomarkers were interpreted based on the Erlangen Score algorithm [32]. The median MMSE score in the group of AD patients was 22 (interquartile range 19–24 points).

The clinical diagnosis of PD was established using EFNS/MDS-ES/ENS recommendations to diagnose Parkinson’s disease [33]. Additionally, the mini-mental state examination (MMSE) was used to assess general cognitive functions. The median of MMSE score in the group of PD patients was 27 (interquartile range 25–28 points).

All MS patients included in the study were diagnosed as relapsing-remitting MS according to MacDonald criteria 2017 [34]. They had a history of at least one clinical attack, and there was no evidence of dissemination in time and atrophy based on the magnetic resonance imaging (MRI) and had the presence of CSF oligoclonal bands (OCBs). The disease severity in patients with MS was assessed using the expanded disability status scale (EDSS). All evaluations rated between 1 and 2 points, indicating an early stage of the disease. All patients with multiple sclerosis have not received any treatment with any disease-modifying drugs at the time of CSF collection. Matching CSF and serum samples from the patients were collected only once during the diagnosis of the disease. Moreover, 32 out of 40 patients had OCBs in the CSF but not in serum (pattern type 2), and 8 had OCBs in CSF and serum, with additional OCBs in the CSF (pattern type 3).

The control group consisting of 20 neurological patients (11 females and 9 males) was carefully selected based on neurological, neuropsychological, and laboratory (blood and CSF) examinations, which allowed the exclusion of the symptoms’ organic background. Additionally, individuals from the control group did not have subjective memory disorders, did not fulfill the MCI criteria, and had no significant changes in the AD biomarkers (such as Aβ1–42, Aβ1–42/Aβ1–40 ratio, Tau and pTau181). The comprehensive inclusion and exclusion criteria for non-demented controls were defined in our previous report [35]. Additionally, simultaneous the concentration of albumin and immunoglobulins in the CSF and serum were assessed to calculate the concentration albumin and immunoglobulins quotients. In the control group, 14 patients had no bands in CSF and serum (pattern type 1), 6 had the same pattern of OCBs in CSF and serum (pattern type 4).

### 2.2. Proteins Measurement

CSF was collected into polypropylene tubes through a lumbar puncture in the L3/L4 or L4/L5 interspace. Immediately after collection, the CSF was centrifuged, aliquoted into polypropylene tubes, and stored at –80 °C until processing. All of the tested proteins assessed in the current study were measured simultaneously, using the same batch of reagents, at the Department of Neurodegeneration Diagnostics, Medical University of Białystok, Poland. CSF samples were run in duplicate. The quantitative analysis of tested proteins was prepared following the manufacturer instructions for each protein. CSF RTN-4 was measured using Human RTN4 (Reticulon 4) ELISA kit (Wuhan, China). CSF Aβ-42 and Aβ-40 were analyzed by using a sandwich ELISA IBL kits (Hamburg, Germany), while CSF Tau and pTau181 were determined using Innotest Fujirebio kits (Europe, Gent, Belgium) following the manufacturer’s instructions. CSF alpha-Synuclein was assessed by alpha-Synuclein ELISA Kit (BioLegend, CA, USA). CSF and serum concentrations of albumin and immunoglobulins were measured using a nephelometer (Optilite; The Binding Site). To assess the integrity of the blood-CSF barrier and intrathecal immunoglobulins production, albumin and immunoglobulins quotients were calculated (Q_Alb_, Q_IgG_, Q_IgA_, Q_IgM_, respectively). Furthermore, the oligoclonal bands were assessed by using isoelectofocusing on agarose gel (Hydragel 3 CSF Isofocusing; Hydrasys; Serbia) following manufacturer protocol.

### 2.3. Statistical Analysis

Statistical analysis was conducted using the *PMCMRplus* package in the statistical software R [36]. The three groups were compared with regards to age, gender distribution, and CSF levels of RTN-4, Aβ-42, Aβ-40, Tau, pTau181. The Shapiro-Wilk test was used to evaluate the distribution of variables. The comparison between four study groups such as AD, PD, MS and the control group was made by using the Kruskal-Wallis test and the post hoc Dwass Steele-Critchlow-Fligner test, which is able to verify in which groups the differences were statistically significant. The results are presented as medians and interquartile ranges. The Spearman correlation coefficient was used for the analysis of relationships between tested variables. P values below 0.05 were regarded as significant. Moreover, the receiver operating characteristic (ROC) curve analysis was used to determine the diagnostic usefulness of the tested proteins as potential biomarkers which could be used to diagnose cognitive disorders.

## 3. Results

### 3.1. Patient Characteristics and CSF Concentrations of RTN-4

**Figure 2:**
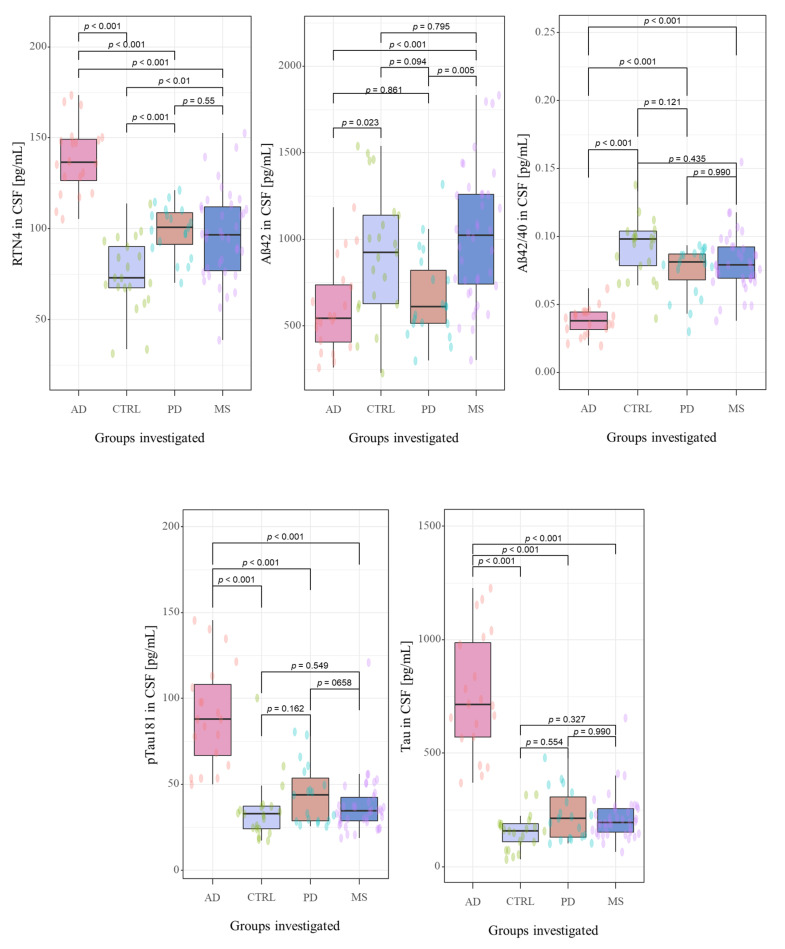
The distribution the CSF concentrations of tested biomarkers in examined groups. Abbreviations: Cerebrospinal fluid (CSF), Reticulon 4 (RTN4), Alzheimer’s disease (AD), Parkinson disease (PD), Multiple sclerosis (MS), Control group (CTRL).

The CSF biomarkers levels in investigated groups were summarized in Table 1 and Figure 2. The study cohorts included 98 subjects, including 77 newly-diagnosed, naïve-treatment patients with neurodegenerative diseases, such as Alzheimer’s disease (n = 20), Parkinson’s disease (n = 19), the neurological condition associated with cognitive decline (multiple sclerosis (n = 38)), and 21 non-demented controls. A detailed description concerning investigated groups was presented in Section 2.1. Study population.

Neurochemical dementia diagnostics biomarkers (NDD) were measured in CSF of all participants of the study. A statistically significant difference between all the study groups for CSF concentrations of RTN-4 (*p* < 0.001, χ^2^ = 47.9), Aβ_1-42_ (*p* < 0.001, χ^2^ = 22.5), Aβ_1-42_/Aβ_1-40_ ratio (*p* < 0.001, χ^2^ = 45.4), tau (*p* < 0.001, χ^2^ = 48.4) and pTau181 (*p* < 0.001, χ^2^ = 37.5) in Kruskal-Wallis test were found.

The post hoc Dwass-Steele-Critchlow-Fligner test was also performed to compare the levels of the tested biomarkers between study groups. CSF concentrations of RTN-4 were significantly higher in patients with AD, PD, and MS than in the control group (*p* < 0.001, W = −7.673; *p* = 0.001, W = 5.305; and *p* = 0.009, W = 4.455; respectively) (Figure 2). Interestingly, comparing the tested proteins concentrations between all investigated groups, only RTN-4 levels differed significantly between almost all study groups (AD vs. PD *p* < 0.001, W = −6.915; AD vs. MS *p* < 0.001, W = −7.195) excluding comparison between PD and MS (*p* = 0.929, W = −0.862) (Figure 2). The highest concentration of this protein was detected in AD patients in comparison with PD and MS groups.

CSF concentration of Aβ_1-42_ was lower in AD and PD groups as compared with MS and Controls, and statistically differentiated between AD and Controls (*p* < 0.001, W = −7.67), PD and MS (*p* = 0.005, W = 4.716;) as well as in the groups AD versus MS (*p* < 0.001, W = 5.703). A similar pattern was observed for Aβ_1-42_/Aβ_1-40_ ratio. The lowest Aβ_1-42_/Aβ_1-40_ ratio was observed in AD patients, and a significant difference was found between AD and Controls (*p* < 0.001 W = 7.377), AD and PD (*p* < 0.001, W = 6.517), as well as in the group AD versus MS (*p* < 0.001 W = 8.421). The CSF levels of total Tau and pTau181 were the highest in the group of patients with AD compared with PD, MS and Controls. Furthermore, statistically significant differences were revealed only for groups AD vs. Controls (*p* < 0.001, W = −7.7; *p* < 0.001, W = −6.12;), AD vs. PD (*p* < 0.001, W = −7.31; *p* < 0.001, W = −6.44) as well as AD vs. MS (*p* < 0.001, W = −8.23; *p* < 0.001, W = −8.14).

### 3.2. Correlation Analysis of RTN-4 and CSF Biomarkers

**Figure 3:**
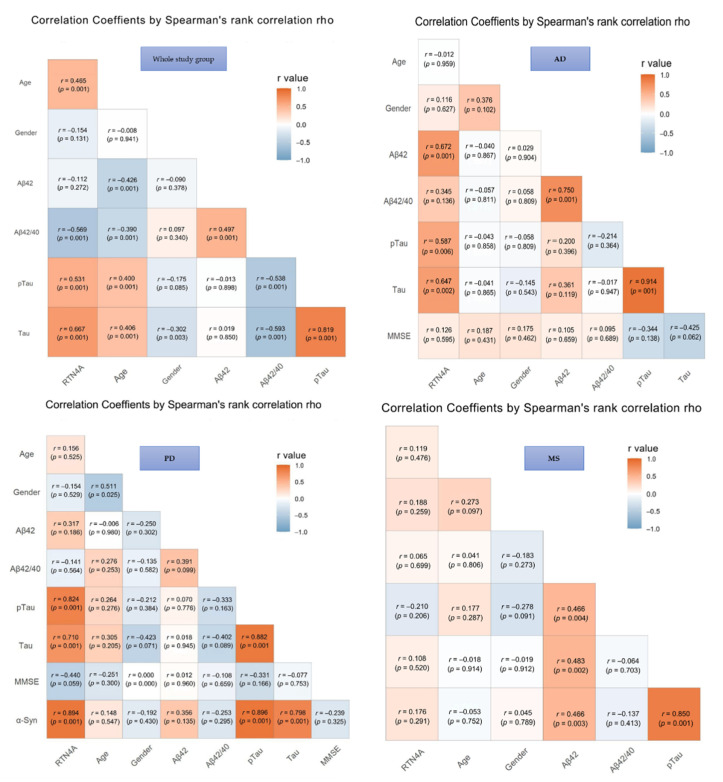
Associations between Reticulon 4 (RTN-4) and tested variables. Abbreviations: Alzheimer’s disease (AD), Parkinson disease (PD), Multiple sclerosis (MS), Control group (CTRL), MMSE – mini-mental state examination.

The results of correlations in the investigated groups were presented in Figure 2. In the total study group significant associations between CSF levels of RTN-4 and classical AD biomarkers, such as Aβ-42/Aβ-40 ratio (*p* < 0.001), tau (*p* < 0.001), and pTau (*p* < 0.001) were found. Moreover, the concentration of RTN-4 correlated also with the severity of cognitive decline assessed based on MMSE scale (*p* < 0.001) and age (*p* < 0.001), but a similar association was not observed with gender in any tested group (Figure 3).

In AD patients, the elevated CSF level of RTN-4 was significantly associated with decreased levels of Aβ-42 (increased *p* < 0.001), Tau and pTau181 concentrations (*p* < 0.002 and *p* < 0.006, respectively). Additionally, a positive correlation between Tau and pTau181 proteins (*p* < 0.001) was also observed.

Increased CSF RTN-4 levels within PD group correlated with higher levels of Tau (*p* < 0.001), pTau181 (*p* < 0.001), and α-synuklein (*p* < 0.001). Moreover, a significant positive correlation was revealed for α-synuklein and Tau and pTau181 proteins (*p* < 0.001 and *p* < 0.001, respectively). The association between tau and pTau181 (*p* < 0.001) was also observed (Figure 3).

In the group of patients with MS exhibited a correlation between Tau and pTau181 (rho = 0.85, *p* < 0.001), Aβ-42 and tau (*p* < 0.003), and pTau (*p* < 0.002). There were no significant correlations between RTN4 and other parameters in the MS group.

### 3.3. Diagnostic Usefulness of RTN-4 as a Candidate Biomarker in the Differential Diagnosis 

The results of the receiver operating characteristic curve (ROC) analysis-areas under the ROC curves (AUC) for RTN-4 and classical biomarkers are presented in Table 2. The analysis of ROC was performed in AD, PD, MS patients compared to Controls and between groups of different NDs. Receiver operating characteristic (ROC) curve analysis demonstrated that CSF levels of RTN4 may significantly discriminate AD patients from controls (AUC = 0.995, *p* < 0.001) and AD from PD (AUC = 0.958, *p* < 0.001) similarly like the commonly used AD biomarkers (tau—AUC = 0.895 and AUC = 0.984, pTau181—AUC = 1.00 and AUC = 0.926, Aβ_1-42_/Aβ_1-40_ ratio—AUC = 0.976 and AUC = 0.932). AUCs values for CSF levels of RTN4 were slightly higher in comparison to classical biomarkers in MS patients as compared to the Controls (AUC = 0.749, *p* < 0.001 vs. tau—AUC = 0.698, pTau181—AUC = 0.566, Aβ_1-42_/Aβ_1-40_ ratio—AUC = 0.653) and in PD versus Controls (AUC = 0.847, *p* < 0.001 vs. tau—AUC = 0.687, pTau181—AUC = 0.639, Aβ_1-42_/Aβ_1-40_ ratio—AUC = 0.739). Additionally, AUCs values for CSF levels of RTN4 were similar as in traditional biomarkers in discrimination MS from AD and PD (Table 2).

## 4. Discussion

At present, in clinical practice, the diagnosis of neurodegenerative diseases is mainly based on a clinical history, which often results in a misdiagnosis, particularly in the early stages of the disease. CSF biomarkers represent a promising diagnostic tool for the early and differential diagnosis of NDs. A combination of multiple CSF biomarkers improves the accuracy of diagnosis and prognosis of these diseases. One of the potential biomarkers in ND seems to be RTN4 protein. The data from the available literature has suggested the relationship between RTNs and pathogenesis of neurodegenerative disease [16,20,37,38]. However, no studies on Alzheimer’s and Parkinson’s diseases have been reported in which the concentration of RTN4 in cerebrospinal fluid was assessed, and there are a few studies with respect to multiple sclerosis. Thus, our study appears to be important to confirm the essential role of this protein in NDs and verify its possible clinical usefulness. According to our best knowledge, this is the first study that assessed and compared the concentrations of RTN4 protein in CSF patients with different NDs. In the light of the above-mentioned fact, it seems to be crucial to get better novel biomarkers of neurodegeneration and their potential clinical usefulness.

In our study, we assessed the concentrations of RTN-4 in CSF patients with two of the most common neurodegenerative diseases, such as AD and PD, subjects with the neurological disease in which cognitive impairment develops (MS), and control group (i.e., individuals without cognitive decline). A significant difference of RTN4 levels were found between AD, PD, MS patients, and controls. However, a higher concentration of RTN-4 was observed in patients with neurodegenerative diseases (AD, PD) than in subjects with MS or the control group. Interestingly, patients with AD presented the highest levels of tested protein compared to other groups of patients, which may indicate the possibility of using it as a potential biomarker. In agreement with our findings are the results of Gil et al., who revealed that RTN-4A protein is constitutively present in the hippocampus of healthy elderly individuals. Interestingly, it is overexpressed in the brain tissue of patients with AD. RTN-4A seems to be a crucial factor promoting amyloid-beta pathology [39]. According to some authors, increased expression of RTN-4A is possibly related to accelerated β-amyloid production and deposition in senile plaques, which may lead to the onset and development of AD [17]. It seems that particularly Nogo-66, a major inhibitory region of RTN-4A, increases the production of Aβ40 and Aβ42 in a dose-dependent manner [20]. Interestingly, other members of RTN4 subfamily (RTN4B and RTN4C) and RTN3 decreased about 30–50% secretion of Aβ40 and Aβ42 [40]. It has been reported that RTN3, and likewise RTN4B and RTN4C, interacting with BACE1 inhibits simultaneously the cleavage of APP and the production of Aβ peptide [41]. Moreover, Nogo-A and NgR1 also play a significant role in simultaneously regulating structural and synaptic plasticity and processes of learning and memory, so it seems possible that overexpression of these proteins may be related to increased risk for cognitive decline observed in AD patients [17,42]. Studies Xiao et al. carried out on APP transgenic mice showed that deletion of RTN-4A improved learning and memory deficits and simultaneously reduced AD-related pathology [19]. The above mentioned studies indicate a direct link between increasing RTN-4 levels and a well-known principal causative factor (Aβ-42) of AD. It seems that increased levels of RTN-4 could influence the disease’s course and probably contribute to disease progression. Therefore, measuring the concentration of this protein could serve as a valuable diagnostic or even prognostic marker.

The role and contribution of RTN-4A in PD pathology are just beginning to be discovered. In the available literature, there are no data concerning the concentration of RTN-4 in CSF of PD. Only a few studies have examined the expression of Nogo-A in the nigrostriatal system, which is particularly implicated in PD [21,43]. mRNA of RTN-4A was found in both the striatum and the substantia nigra (SN), whereas RTN-4A protein was shown in large neurons in the caudate-putamen [44,45]. Schawkat et al. revealed a significant reduction of RTN-4A positive dopaminergic neurons in the first week after the lesion and the same tendency thereafter. Furthermore, initially neurons co-expressing Nogo were more susceptible for lesions, but at the later phase of the disease, a higher amount of the surviving dopaminergic neurons co-expressed Nogo-A [21]. This may indicate that RTN-4A expression is regulated in response to the injury and acts as a neuroprotective factor on dopaminergic neurons co-expressing Nogo-A in the course of the Parkinson’s disease. Interestingly, recent studies also indicate the role of RTN4A in the pathology of mild cognitive impairment in PD (PDMCI). Wang et al., in studies on the animal model, found that RTN4A through the sphingosine-1-phosphate receptor 2 (S1PR2) and the RhoA/ROCK signaling pathway may inhibit vascular remodeling, causing spatial memory and behavioral impairment. Moreover, downregulation of Nogo-A led to alleviation of the cognitive impairment and microvascular dysfunction as well as the inhibition of the expression of S1PR2 and the RhoA/ROCK signaling pathway [46]. It seems that additional studies are necessary to elucidate the function of RTN-4A in dopaminergic neurons, especially concerning its signaling pathways and co-receptors.

In agreement with our findings, other studies have also demonstrated the increased RTN4 levels in the chronic active demyelinating lesions of brain tissues, cerebrospinal fluid, and blood patients with multiple sclerosis [25,26,27,28]. Notably, an elevated level of RTN4A was observed in CSF patients with both types of the disease: remitting-relapsing MS (RR-MS) and secondary progressive MS (SP-MS). Moreover, the overexpression of RTN4A was found in the whole spectrum of the disease (at the onset of MS, in a long-lasting and advanced stage of disease), which may indicate the possibility of using it as a potential biochemical marker of the disease [25]. However, opposing data are also available [47]. Studies of Jitoku et al. in patients with schizophrenia confirm the role of RTN4A in myelination. The authors found a positive correlation between negative psychotic symptoms and RTN4A concentrations, which supports the theory that RTN4A signaling regulates structural and synaptic plasticity [48]. It is suggested that antibodies neutralizing inhibitory effects of Nogo-A may serve as a potential therapeutic target. Studies on the MS mouse model EAE revealed that blocking Nogo-A receptors ameliorated the disease course, accelerated functional recovery, and increased axonal sprouting and remyelination [15,29]. It seems interesting to verify the relationship of the protein concentrations with clinical outcomes and therapeutic effects.

Our study has shown a significantly higher concentration of RTN4 in patients with MS than the controls but a lower concentration than in other groups (AD and PD). Given that RTN-4A protein is possibly a major component of myelin sheaths in the central nervous system (CNS) and a key inhibitor of neuron regeneration, we expected the highest levels of RTN-4 in CSF of patients with MS. The highest level in AD is perhaps due to the fact that the process of CNS cell damage leading to neurodegeneration in patients with newly diagnosed MS is not yet as advanced as in people with full-blown AD disease. In the course of MS, it is the damage to the vital nuclear cells for the functioning of the CNS, such as neurons, oligodendrocytes, and astrocytes, which is responsible for the most significant progression of patients’ disability. Maybe in a more advanced stage of disease, the concentration of the RTN4 in patients with MS will increase. Therefore, it could be essential to study the dependence of RTN4 concentration on the disease duration and the probability of using this protein as a potential prognostic or even predictive biomarker. Moreover, it seems to be the approach measuring the level of RTN4 protein not only in various neurological diseases manifested by cognitive impairment but also in different stages of advancement of cognitive decline. So that it can be verified to what extent the secretion of these proteins may reflect their direct relationship with cognitive functions. Our research showed a significant difference in the concentrations of the tested protein between AD and PD as well as AD and MS, which indicates the possibility of using it as a biomarker in the differential diagnosis of cognitive disorders. However, we did not observe such a difference between the groups of patients with PD and MS, which may suggest some relationship with the pathomechanism of these diseases.

In the present study, we assessed the diagnostic usefulnes of CSF RTN4 levels as a clinical biomarker enabling discrimination of NDs on the basis of ROC curves. The AUC values of proposed, novel biomarker were similar to the classical AD biomarkers when were compared AD patients with controls, PD and MS. However, AUC values for discriminating between controls and PD and MS patients were slightly higher then the AUCs for the classical biomarkers. According to our best knowledge, there is a lack of studies in the available literature evaluating the diagnostic value of this biomarker in NDs. Therefore, there is a need to confirm the clinical value of RTN4 in future studies, particularly in combination with the existing biomarkers or imaging tools.

The pathological role of tau in tauopathies, such as Alzheimer’s disease, Parkinson’s disease, Pick’s disease, and Huntington’s disease, is well known [49,50]. Therefore, we also verified the possible relationship of Tau proteins with RTN4. As we expected, our study revealed the correlation between RTN-4 protein and Tau, as well as pTau181 proteins in the whole study group (patients with AD and PD). This was in line with an earlier report, which showed that inhibition of the Nogo/NgR pathway alleviates pathological features such as amyloid plaques and phosphorylated tau in APP/PS1 mice. The authors demonstrated that the inhibition of the Nogo/NgR signaling could influence tau phosphorylation through inhibiting the activity of GSK3β and thereby potentially affect the accumulation of NFTs in the AD mice brain [51]. Studies on neuroblastoma 2a (N2a) cells and primary cortical neurons also revealed the Nogo-66 might influence the levels of Tau proteins. Nogo-66 transiently increased the total tau protein level and persistently decreased the level of p-S262 tau (tau phosphorylated at serine 262). Furthermore, the tau overexpression can save the Nogo-66-induced inhibition of neurite outgrowth in vitro [52].

Moreover, this study showed an age-dependent change in RTN-4 protein level in CSF of all neurological patients. We observed increased levels of the RTN4 protein in CSF patients with neurological diseases. Previous studies have shown lowering expression of RTN-4 protein in developing mice [53,54]. The effect of age on RTN4 level was showed by Kumari et al. in mouse brain cerebrum. The authors revealed a decrease in RTN4A protein in aging but no age-related alterations of RTN4A mRNA, as well as NgR1 protein and its mRNA levels [55]. In line with these findings is the study Mingorace et al., who reported that RTN4A protein expression decreased from the neonate to the adult stages in the developing hippocampus [54]. This result is also supported by the studies of Gil et al., where they have demonstrated a decline in the RTN4A protein expression in telencephalon from embryo to adult stages [53]. It is well known that RTN4A protein is abundant in the highly plastic areas of mature CNS [11]. Additionally, RTN4A and its receptor NgR1 regulate structural plasticity [42] and play a crucial role in the process of learning and memory [13,42], which are impaired with normal aging. The downregulation of the RTN4A/NgR1 pathway is a physiological component of plastic changes needed for long-term learning [55]. Considering that expression of RTN4A protein is downregulated and NgR1 is unaltered in physiological conditions with brain aging, we may speculate that increase of RTN4A protein levels in patients with neurological diseases is a part of a pathological mechaniMS activated in the central nervous system (CNS), which may gradually lead to cognitive decline. In agreement with this notion, and partially our findings, is study Vanguild et al. that demonstrated upregulation of NgR1 in aged cognitively impaired, but not age-matched cognitively normal rats [56]. We also found the correlation with MMSE scale but only in the whole study group therefore, this requires additional studies. In the light of the above-mentioned facts, the increase of the RTN-4 level in patients with neurological disease may be considered a negative predictive factor of cognitive impairment.

Given the fact that there is still little evidence that an increase of RTN-4 levels observed in AD, PD or MS has a direct role in disease progression, the question arises whether such a role is conceivable. Moreover, how could aberrant or a higher level of RTN-4 protein itself lead to neuropathology? To come up with plausible answers, a good understanding of RTN4-mediated inter and intracellular signaling pathways is necessary.

## 5. Conclusions

In conclusion, detecting neurodegeneration biomarkers may facilitate early diagnosis, improve differential diagnosis, and increase prognostic accuracy. The results of our study confirm the association of RTN4 with crucial for some neurodegenerative disease processes, including amyliodopathy, neurodegeneration, and α-synucleinopathy. Moreover, the comparative analysis of CSF RTN4 levels revealed significantly higher concentrations in patients with neurodegenerative diseases, particularly with AD. Interestingly, only the increased concentration of RTN4 protein al-low for differentiation of almost all study groups with diagnostic accuracy similar to classical biomarkers. Therefore, it seems that these preliminary CSF results may support the potential clinical usefulness of RTN4 in the differential diagnosis of NDs. However, our findings need to be clarified by additional studies on a larger group of patients.

## Figures and Tables

**Table 1:** CSF biomarkers levels in the investigated groups.

Biomarkers	AD	PD	MS	CTRL	Kruskal-Wallis Test	Significance (Dwass Steel Critchlow-Fligner Test)					
	Median (Interquartile Range)	Median (Interquartile Range)	Median (Interquartile Range)	Median (Interquartile Range)		AD vs. CTRL	PD vs. CTRL	MS vs. CTRL	AD vs. PD	PD vs. MS	AD vs. MS
**RTN-4 (pg/mL)**	137	101	97	73	<0.001	<0.001	0.001	0.009	<0.001	0.929	<0.001
	(126–149)	(91–109)	(77–112)	(68–90)							
**Aβ-42 (pg/mL)**	544	612	1023	925	<0.001	0.023	0.094	0.795	0.861	0.005	<0.001
	(407–736)	(515–821)	(741–1260)	(627–1084)							
**Aβ-42/Aβ-40 ratio**	0.038	0.081	0.079	0.095							
	(0.032–0.045)	(0.068–0.087)	(0.069–0.092)	(0.070–0.102)	<0.001	<0.001	0.121	0.435	<0.001	0.990	<0.001
**Tau (pg/mL)**	714	213	194	167	<0.001	< 0.001	0.554	0.327	<0.001	0.990	<0.001
	(571–987)	(130–306)	(152–255)	(110–190)							
**pTau181 (pg/mL)**	88	44	35	33	<0.001	< 0.001	0.162	0.549	<0.001	0.658	<0.001
	(67–108)	(29–54)	(29–42)	(24–37)							

**Table 2:** AUC of tested proteins in compared groups.

Variable Tested	ROC Criteria in AD Compared to CTRL			
	AUC	SE	95% C.I. (AUC)	*p*
RTN4	0.995	0.006	0.9839–1	<0.001
Aβ42	0.762	0.077	0.6103–0.9135	<0.001
Aβ42/40	0.976	0.024	0.9283–1	<0.001
Tau	0.895	0.066	0.7663–1	<0.001
pTau181	1.00	0.00	1–1	<0.001
**ROC criteria in** **PD compared to CTRL**				
RTN4	0.847	0.061	0.7267–0.9675	<0.001
Aβ42	0.719	0.084	0.5546–0.884	0.005
Aβ42/40	0.739	0.083	0.5159–0.8575	0.002
Tau	0.687	0.091	0.5159–0.8575	0.063
pTau181	0.639	0.087	0.4607–0.8175	0.016
**ROC criteria in** **AD compared to PD**				
RTN4	0.958	0.034	0.9024–1	<0.001
Aβ42	0.574	0.093	0.3896–0.7578	0.216
Aβ42/40	0.932	0.044	0.8441–1	<0.001
Tau	0.984	0.021	0.957–1	<0.001
pTau181	0.926	0.045	0.85–1	<0.001
**ROC criteria in** **MS compared to CTRL**				
RTN4	0.749	0.065	0.6226–0.8762	<0.001
Aβ42	0.554	0.081	0.3953–0.7125	0.253
Aβ42/40	0.653	0.078	0.4936–0.8122	0.030
Tau	0.698	0.073	0.5547–0.8413	0.003
pTau181	0.566	0.083	0.4023–0.7293	0.215
**ROC criteria in** **AD compared to MS**				
RTN4	0.909	0.038	0.8346–0.9838	<0.001
Aβ42	0.824	0.054	0.7144–0.9343	<0.001
Aβ42/40	0.979	0.017	0.9488–1	<0.001
Tau	0.968	0.021	0.9161–1	<0.001
pTau181	0.963	0.023	0.9163–1	<0.001
**ROC criteria in** **PD compared to MS**				
RTN4	0.550	0.080	0.4003–0.6994	0.257
Aβ42	0.773	0.062	0.645–0.9007	<0.001
Aβ42/40	0.525	0.081	0.3147–0.6354	0.380
Tau	0.501	0.082	0.317–0.6802	0.494
pTau181	0.594	0.078	0.4262–0.7621	0.136

## Data Availability

The data presented in this study are available on request from the corresponding author. Key data are stated in the text.
